# Dosimetric evaluation study of IMRT and VMAT techniques for prostate cancer based on different multileaf collimator designs

**DOI:** 10.1007/s00411-022-01011-2

**Published:** 2022-12-28

**Authors:** Mohamed M. Fathy, Belal Z. Hassan, Reem H. El-Gebaly, Maha H. Mokhtar

**Affiliations:** 1 Department of Biophysics, Faculty of Science, Cairo University, Giza, Egypt; 2 Department of Radiotherapy, Al-Ziraeyeen Hospital, Cairo University, Giza, Egypt; 3 Department of Radiotherapy and Nuclear Medicine, Medical Physics Unit, National Cancer Institute, Cairo University, Giza, Egypt

**Keywords:** Multileaf collimator, Free flattening filter (FFF), Flattening filter (FF), Volumetric modulated arc therapy (VMAT), Intensity-modulated radiotherapy (IMRT)

## Abstract

The hypofractionated radiotherapy modality was established to reduce treatment durations and enhance therapeutic efficiency, as compared to conventional fractionation treatment. However, this modality is challenging because of rigid dosimetric constraints. This study aimed to assess the impact of multi-leaf collimator (MLC) widths (10 mm and 5 mm) on plan quality during the treatment of prostate cancer. Additionally, this study aimed to investigate the impact of the MLC mode of energy on the Agility flattening filter (FF), MLC Agility-free flattening filter (FFF), and MLCi2 for patients receiving hypofractionated radiotherapy. Two radiotherapy techniques; Intensity Modulated Radiotherapy (IMRT) and Volumetric Modulated Arc Radiotherapy (VMAT), were used in this research. In the present study, computed tomography simulations of ten patients (six plans per patient) with localized prostate adenocarcinoma were analyzed. Various dosimetric parameters were assessed, including monitor units, treatment delivery times, conformity, and homogeneity indices. To evaluate the plan quality, dose-volume histograms (DVHs) were estimated for each technique. The results demonstrated that the determined dosimetric parameters of planning target volume (PTV)p (such as D mean, conformity, and homogeneity index) showed greater improvement with MLC Agility FF and MLC Agility FFF than with MLCi2. Additionally, the treatment delivery time was reduced in the MLC Agility FF (by 31%) and MLC Agility FFF (by 10.8%) groups compared to the MLCi2 group. It is concluded that for both the VMAT and IMRT techniques, the smaller width (5 mm) MLCs revealed better planning target volume coverage, improved the dosimetric parameters for PTV, reduced the treatment time, and met the constraints for OARs. It is therefore recommended to use 5 mm MLCs for hypofractionated prostate cancer treatment due to better target coverage and better protection of OARs.

## Introduction

Prostate cancer is one of the most common types of tumours among males worldwide (Alongi et al. 2013). External beam radiation therapy has historically been a mainstay of treatment for a large portion of these patients. Generally, the goal of radiation therapy is to provide a high radiation dose to the targeted tumour while simultaneously avoiding the healthy surrounding organs at risk (OARs) (Chae et al. 2016).

Intensity-modulated radiation treatment (IMRT) techniques were introduced to replace traditional 3D-conformal radiation therapy (3D-CRT) techniques, and this change in technique resulted in significantly greater dose conformity, sparing of the OARs, and lower radiation-induced toxicity (Vergeer et al. 2009); Van et al. 2008; (Nutting et al. 2009); (Holt et al. 2013). The fundamental benefit of IMRT is that it can deliver a specified dose of radiation to cancer target volumes with complicated geometries. Another unique characteristic of IMRT is that it can utilize dynamic multileaf collimators (DMLCs) to administer various doses to different target volumes within a single plan. Because the dynamic MLC-IMRT leaves are in constant motion during therapy for each field (Jothybasu et al. 2009), the treatment time for each field is reduced. Each pair of opposing MLC leaves is swept across the target volume at a fixed beam angle while the speed and distance between leaves vary, and this action delivers the desired radiation intensity to a specific spot (Jothybasu et al. 2009); (Clark et al. 2002). The latest generation of IMRT techniques, volumetric modulated arc therapy (VMAT), has recently become widely available. In comparison to static-beam IMRT, rotating VMAT is designed to reduce treatment times while maintaining or improving plan quality (Bedford 2009); (Holt et al. 2013). Both IMRT and VMAT depend on the use of multileaf collimators (MLCs) for radiation therapy.

VMAT technology is a popular radiation delivery approach for prostate cancer treatment because it takes less time and uses fewer monitor units (MUs) than IMRT (Li et al. 2018). VMAT depends on the manipulation of gantry rotation, MLC movement, and dose rate modulation. To provide an optimum dose distribution, and thus a stronger therapeutic impact, two or more VMAT arcs are typically used to assure intricate target forms, target volumes, and differing dose prescription (Chae et al. 2016); (Li et al. 2018).

Clinical use of linear accelerators (linacs) with free flattening filter (FFF) is now possible because of VMAT and IMRT approaches, which may offer a substantially higher dose rate than in the typically used flattening filter (FF). The primary advantage of the FFF mode in radiation therapy is that it increases the dose rate while reducing head scattering and radiation leakage, resulting in an improved delivery efficiency and an increase in MUs, which helps to reduce the dose to OARs (Cakir et al. 2019; Arslan et. al.^2020^).

MLC is the most appropriate tool for beam shaping, and it is specific to each linear accelerator head type. Each type of MLC has specific characteristics, such as the leaf width, maximum leaf speed, minimum gap between opposing leaves, and inter-digitations abilities (Kantz et al. 2015). Therefore, the main purpose of this research was to investigate the dosimetric impact of different MLC designs on patients with localized prostate cancer by making comparisons between VMAT and IMRT techniques. This study was also designed to demonstrate any difference between FF and FFF plans at 5 mm leaf width of MLCs employing VMAT and IMRT, and the potentially resulting additional benefits for patients treated at different sites.

## Patients and methods

### Treatment planning

Computed tomography (CT) simulations of ten patients with prostate cancer were selected for this study. Prior to CT simulation, patients were instructed to have a comfortably full bladder and an empty rectum. Three radio-opaque reference markers were then placed on the patient skin. Serial CT cuts of the abdomen and pelvis were obtained with 2.5 mm slice thickness (Cuccia et al. 2018). CT scans were simulated by a GE Light Speed Scanner (GE Health care Diagnostic Imaging). Images were then transferred to the focal contouring station for the delineation of the target (clinical target volume (CTV) and planning target volume (PTV)) and risk structure. The International Commission on Radiation Units and Measurements (ICRU) has been involved in an effort to improve collaboration in radiation treatment reporting. In a series of Reports (no. 29,38, 50, 58, 62, and 71) (Purdy et al. 2004) (Landberg et al. 1999) (Born et al. 2006) (Stroom et al. 2002) (Berthelsen et al. 2007), recommendations for defining different volumes and dose specification points in radiotherapy were developed (Menzel 2014). The entire rectum, bladder, femoral heads, and penile bulb should be delineated according to the Radiation Therapy Oncology Group (RTOG) guidelines for a typical male pelvis (Gay et al. 2012).

The entire prostate gland was defined as Clinical Target Volume 60 (CTV), and the proximal 10 mm of the seminal vesicles were also included. The planning target volume (PTV60) was created with 7 mm expansion in all directions, with the exception of 4 mm posteriorly. The pelvic lymph node was defined as CTV44, which includes the distal common iliac, external, and internal iliac and obturator vessels. PTV44 was created with 7 mm in all directions. All of the plans were generated on the Monaco planning system (Version 5.11.02). Hypofractionated radiation therapy dose was delivered to the prostate (PTV60) with 60 Gy/20 fractions including a simultaneous integrated boost (SIB) to pelvic nodes (PTV44) 44 Gy/20 fractions.

### Properties of multileaf collimators

This study investigated two linear accelerator head designs of MLC parts: one with Agility MLC parts (Elekta Versa HD) including different modes of energies typically used in modern linear accelerators (FF and FFF), and the other with MLCi2 parts (Elekta Synergy). Each type of MLC has unique characteristics in terms of leaf width, maximum speed, and minimum gap between opposing leaves as well as inter-digitation capabilities (Kantz et al. 2015).

The Elekta Agility MLC (Elekta AB, Stockholm, Sweden) beam pattern included 80 pairs of leaves, with each leaf pair measuring 5 mm wide, projected at the iso-center. The maximum field size was 40 × 40 cm. Leaves had a speed width of 3.5–6.5 cm/s and a minimum gap of 3 mm between opposite leaves joined with a dynamic leaf index, and the leaves can inter-digitize. Under the leaves, there was no auto-tracking backup diaphragm jaw (Table 1) (Ruschin et al. 2016); (Bedford et al. 2013).

**Table 1:** Multileaf collimator (MLC) parameters for Agility MLC and MLCi2 designs

MLC parameters	MLCi2	Agility
Leaf width	10 mm	5 mm
Leaf speed	2 cm/s	6.5 cm/s
Minimum leaf gap	5 mm	3 mm
Inter-digitation	−/ +	+
Backup jaws	Yes	No

In comparison, the MLCi2 had 40 pairs of leaves with 10 mm leaf width at the iso-center. The maximum field size was 40 × 40 cm. Leaves had a speed of about 2 cm/s and had a minimum gap of 5 mm between opposite leaves. Leaves had auto-tracking backup diaphragms beyond them, and backup jaws moved under the treatment to reduce leakage (Table 1). The maximum space between leaves in the same leaf directory was 32.5 cm, the leaves were able to move over the central axis up to a distance of 12.5 cm, and leaves allowed to inter-digitize (Kantz et al. 2015). Table 1 summarizes the differences between the MLC types.

### Dosimetric and plan evaluation

In this study, all plans were designed to compare plan quality and dose distribution among different types of MLC (Agility, MLCi2). For each patient, six plans were performed (VMAT: Agility FF, FFF and MLCi2) (IMRT: Agility FF, FFF and MLCi2) with an energy corresponding to a linac voltage of 6 MV and a fixed number of beams, arcs, angles, segments, and constraints. All plans were evaluated based on DVH. Ninety-five percent of the prescribed doses were accepted to cover ≥ 95% of the PTV, and all patients on the protocol were treated with a VMAT and IMRT technique (6 MV) requiring minimum PTV coverage of V95% prescription dose (60 Gy for prostate targets, 44 Gy for lymph nodes), reported as RTOG (Neto et al. 2015). Also, dosimetric indices such as conformity index (CI), homogeneity index (HI), and normalized dose contrast (NDC) of the PTV were evaluated on the basis of the International Commission on Radiation Units and Measurements report 62 (ICRU). The CI was defined as the volume of the PTV receiving the prescribed dose divided by the volume of the PTV. The ideal value for CI is one. The HI was calculated as follows (Eq. 1):

1 $$\mathrm{HI}=\frac{(\mathbf{D}2\mathbf{\%}-\mathbf{D}98\mathbf{\%})}{\mathbf{P}\mathbf{r}\mathbf{e}\mathbf{s}\mathbf{c}\mathbf{r}\mathbf{i}\mathbf{b}\mathbf{e}\mathbf{d}\mathbf{d}\mathbf{o}\mathbf{s}\mathbf{e}}$$

where D2% is the dose received by 2% of the target volume, D98% is the dose received by 98% of the target volume. A lower HI value indicates better dose homogeneity, and its optimal value is zero. The NDC value is defined by Eq. 2.

2 $$\mathrm{NDC}=\frac{\mathbf{A}\mathbf{c}\mathbf{t}\mathbf{u}\mathbf{a}\mathbf{l}\mathbf{D}\mathbf{C}}{\mathbf{I}\mathbf{d}\mathbf{e}\mathbf{a}\mathbf{l}\mathbf{D}\mathbf{C}}$$

where Actual DC is equal to the mean dose of (PTV60) divided by the mean dose of (PTV44), while the Ideal DC is calculated from the ratio between the prescribed doses of (PTV60) to the prescribed dose of (PTV44). This value was used to compare the dose gradient with an optimum value equal one.

Dose-volume constraints for the hypofractionated prostate radiotherapy protocol used in the present study were as follows: for the rectum—V60 Gy < 15%, V56 Gy < 25%, V52 Gy < 35%, and V48 Gy < 50%; for the bladder: V60 Gy < 25%, V56Gy < 35%, and V52 Gy < 50%; for the penile bulb: mean dose of < 42 Gy; for the femoral heads: maximum dose (max dose) < 45 Gy; and for the small bowel bag: V45 Gy < 200 mL and D5 mL < 60 Gy (Ruschin et al. 2016).

### Statistical analysis

The data were analyzed using the Statistical Package of Social Science (version 26). Data were presented as mean ± standard deviation. The different letters (A, B, C) indicate significant differences between the means of each parameter. The comparison between groups was performed using the Friedman test followed by the post hock test for pairwise comparison between groups. All tests were two-tailed, with a *P* value of < 0.05 considered significant.

## Results

**Fig. 1:**
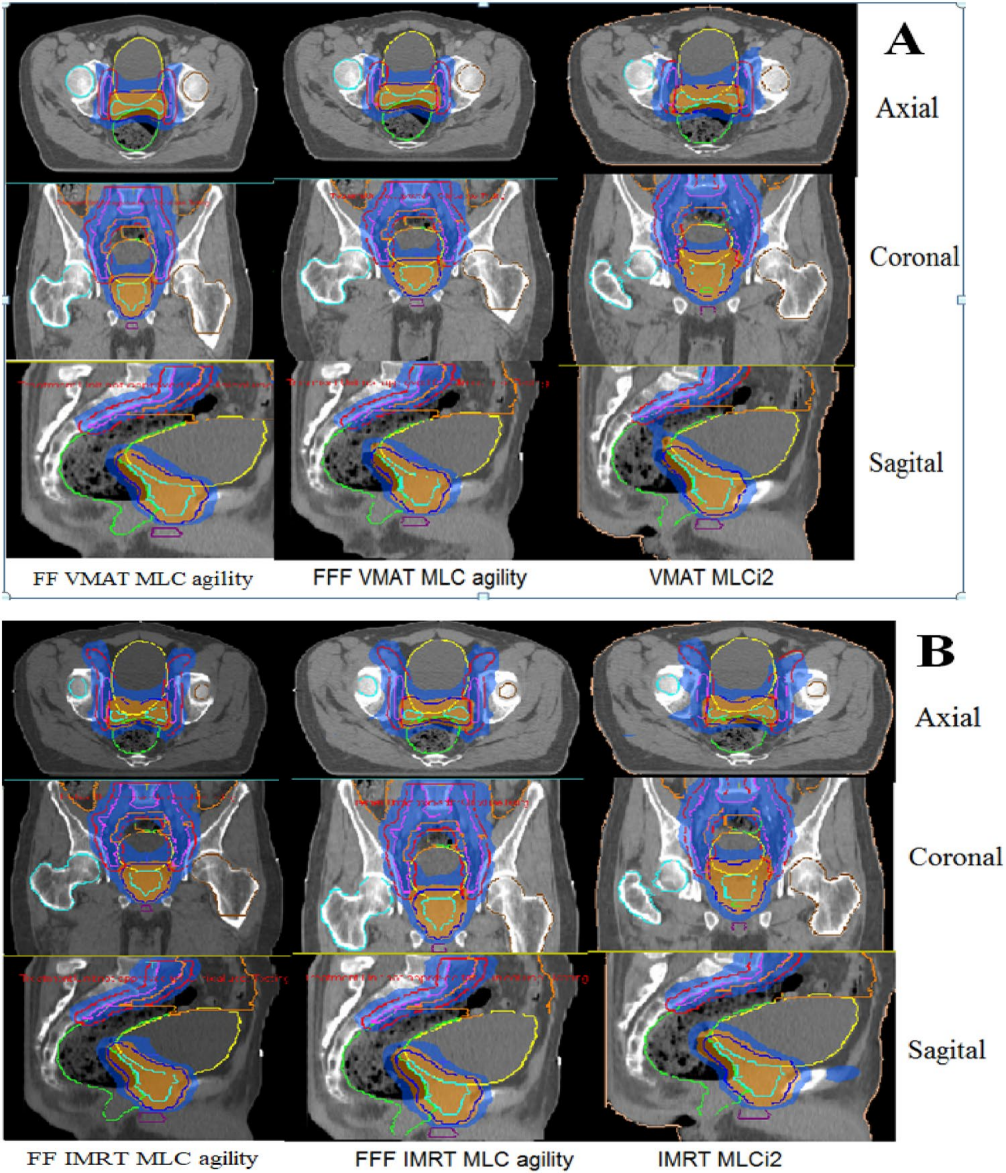
Dose distribution in lymph nodes and prostate for **A** VMAT and **B** IMRT plans, for different multileaf collimators (MLCs), respectively

**Fig. 2:**
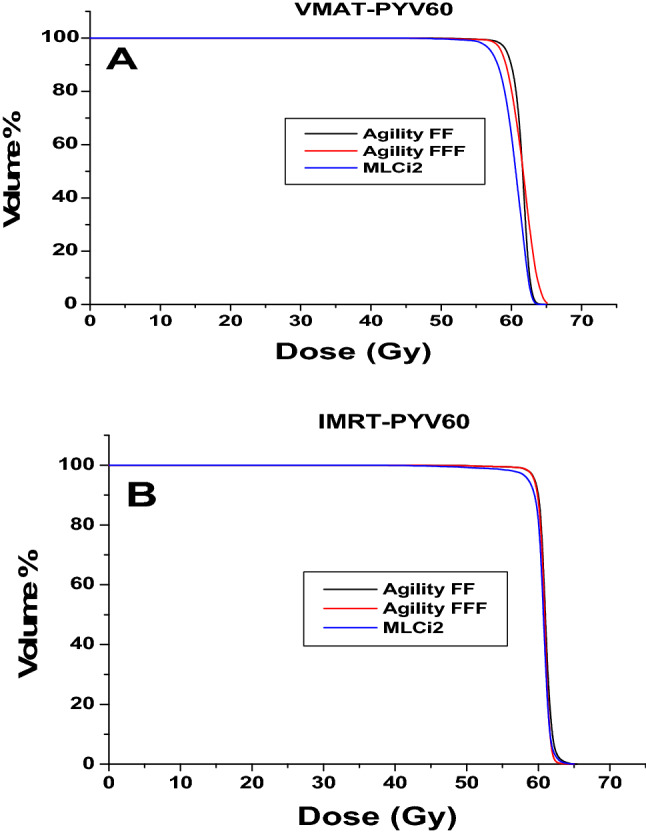
Comparison of **A** VMAT and **B** IMRT plans with dose-volume histograms for PTV 60

For each patient, six plans were generated, and the results were then divided into two sets. The first result set was VMAT at different MICs (MLCi2, Agility (FFF), and Agility (FF)), while the second set included corresponding IMRT results. The acceptance criteria achieved in all plans with VMAT and IMRT techniques depend on the PTVs and OARs. Figure 1A, B show the dose distribution for VMAT and IMRT plans with different MLC designs for PTV60 and PTV44. Also, Fig. 2A, B reveal the PTV60 DVH of VMAT and IMRT with different MLC. Additionally, Tables 2, 3 summarize the dosimetric DVH parameters of the PTVs and OARs with different MLC types for VMAT plans.

**Table2:** Dosimetric parameters of planned treatment volumes (PTVs) for VMAT plans (mean ± standard deviation, *n* = 10)

	VMAT technique on different types of MLC		
	Agility FF	Agility FFF	MLCi2
Parameters (PTV60)			
D2% (Gy)	63.8 ± 0.7 (A)	64 ± 0.73 (A)	63.3 ± 0.55 (A)
D98% (Gy)	58.3 ± 1.25 (A)	57.9 ± 1.4 (A)	56.9 ± 1.1 (B)
D95% (Gy)	59.3 ± 0.7 (A)	58.9 ± 0.88 (A)	58.2 ± 0.7 (B)
D50% (Gy)	61.7 ± 0.51 (A)	61.6 ± 0.68 (A)	61.1 ± 0.4 (B)
D MAX (Gy)	66 ± 0.98 (A)	66.2 ± 0.75 (A)	65.6 ± 0.87 (A)
D min (Gy)	51.3 ± 5 (A)	50.8 ± 5.6 (A)	47.63 ± 5.9 (B)
D mean (Gy)	61.5 ± 0.53 (A)	61.5 ± 0.68 (A)	60.9 ± 0.4 (B)
Homogeneity (HI)	0.09 ± 0.02 (A)	0.095 ± 0.01 (A)	0.11 ± 0.02 (B)
Conformity (CI)	0.89 ± 0.054 (A)	0.85 ± 0.1 (A)	0.78 ± 0.1 (B)
Parameters (PTV44)			
D2% (Gy)	56.5 ± 0.91 (A)	56.4 ± 0.1 (A)	56.2 ± 0.77 (A)
D98% (Gy)	42.8 ± 0.5 (A)	42.5 ± 1.1 (A)	42.4 ± 0.67 (A)
D95% (Gy)	43.6 ± 0.4 (A)	43.3 ± 0.88 (A)	43.24 ± 0.56 (A)
D50% (Gy)	46.2 ± 0.3 (A)	46.1 ± 0.4 (A)	45.97 ± 0.19 (A)
D MAX (Gy)	63.04 ± 1.1 (A)	63.1 ± 0.87 (A)	62.97 ± 1.83 (A)
D min (Gy)	37.1 ± 2.99 (A)	38.95 ± 6.4 (A)	35.49 ± 1.92 (B)
D mean (Gy)	46.7 ± 0.2 (A)	48.1 ± 4.99 (A)	46.46 ± 0.31 (A)
Homogeneity (HI)	0.3 ± 0.01 (A)	0.31 ± 0.03 (A)	0.31 ± 0.02 (A)
Conformity (CI)	0.92 ± 0.02 (A)	0.89 ± 0.07 (A)	0.89 ± 0.05 (A)
NDC	0.97 ± 0.01 (A)	0.95 ± 0.08 (B)	0.96 ± 0.01 (C)

For each parameter (D2%, D98%, D95%) there are statistically significant differences between different modes of energy (Agility FF, Agility FFF, and MLCi2) when indicated with different letters (A, B, and C); i.e., different letters (A, B, C) represent significant differences between the means for each parameter

*D2%* is the dose received by 2% of the PTV, *D98%* is the dose received by 98% of the PTV, *D95%* is the dose received by 95% of the PTV, *D50%* is the dose received by 50% of the PTV, *Dmax* the maximum dose of PTV, *Dmin* the minimum dose of PTV, *Dmean* average dose of PTV

*P* ≤ 0.05 was defined as statistically significant

**Table 3:** Comparison of VMAT plans for organs at risk (OARs) with dosimetric parameters

Parameters of OARs	VMAT technique on different types of MLC		
	Agility FF	Agility FFF	MLCi2
Bladder			
V60Gy < 25%	9.6 ± 1.19 (A)	9 ± 1.13480 (A)	7.2 ± 1.7 (B)
V56Gy < 35%	16.7 ± 1.3 (A)	16.1 ± 1.3 (A)	15.6 ± 1.9 (A)
V52Gy < 50%	22 ± 1.3 (A)	21.3 ± 1.5 (A)	21.6 ± 1.3 (A)
D mean (Gy)	36.7 ± 5.8 (A)	37.4 ± 5.6 (A)	37.7 ± 6.2 (A)
D max (Gy)	65 ± 0 (A)	65 ± 1.2 (A)	64.2 ± 1.3 (B)
Rectum			
V60Gy < 15%	4.2 ± 3.3 (A)	3.95 ± 3.8 (A)	2.96 ± 3.13 (A)
V56Gy < 25%	9.6 ± 4.2 (A)	10 ± 5.2 (A)	9.1 ± 5.4 (A)
V52Gy < 35%	14.6 ± 4.9 (A)	15.1 ± 6.1 (A)	14.8 ± 6.6 (A)
Mean dose (Gy)	37.1 ± 3 (A)	36.6 ± 3.1 (A)	37.2 ± 3.5 (A)
Maximum dose (Gy)	63.8 ± 1.5 (A)	63.5 ± 1.5 (A)	63.1 ± 1.2 (A)
Femoral (MAX dose) < 45 Gy			
Right	39.3 ± 2.6 (A)	39.2 ± 2.7 (A)	39.2 ± 3.1 (A)
Left	39 ± 2.1 (A)	39 ± 2.1 (A)	39.8 ± 2.8 (A)
Bowel bag			
V45Gy < 200 ml	76.2 ± 53.1 (A)	67.4 ± 59.2 (A)	57.9 ± 46.8 (B)
D (5 ml) < 60 Gy	48.4 ± 1.2 (A)	48.1 ± 1.5 (A)	47.7 ± 1.3 (B)
Penile bulb			
D (mean) < 45 Gy	23.3 ± 10 (A)	22.7 ± 9.75 (A)	24.7 ± 8.3 (A)

For each parameter, there are statistically significant differences between different modes of energy (Agility FF, Agility FFF, and MLCi2) when indicated with different letters (A, B)

OARs parameters; *VX Gy < X%* percent volume of OARs receiving a dose of x Gy in less than x% of the volume, *Dmax* maximum dose, *Dmean* average dose, *VX Gy < Xml* volume of OARs in milli litter (ml) receiving a dose of x Gy is less than x ml, *D (Xml) < X Gy* no more than X Gy received by X ml of the volume

*P* ≤ 0.05 was defined as statistically significant. For details see text

### VMAT dosimetric parameters of PTVs and OARs

Regarding PTV60, the results revealed that there were no significant differences for D2% between VMAT techniques with different MLC types (MLCi2, Agility FFF and Agility FF). The remaining parameters (D98%, D95%, D50%, Dmin and Dmean) showed no significant difference between Agility FF and FFF. In contrast, there were statistically significant differences between Agility (FFF and FF) and MLCi2, as shown in Table 2. The results also revealed that CI and HI had the optimal values for Agility FF (0.89, 0.09) and FFF (0.85, 0.095) compared with MLCi2 (0.78, 0.11), respectively (Table 2). There were statistically significant differences between Agility (FFF and FF) and MLCi2 for CI and HI.

For PTV44, VMAT plan dosimetric parameters showed non-significant differences between Agility FF, FFF and MLCi2, with the exception of Dmin (Gy) for which there was a significant difference with agility FF and FFF compared to MLCi2. The OARs in Table 3 achieved the criteria that have been set in the planning system for all plans using the VMAT technique with different types of MLCs and different modes of energy. In terms of bladder dose, MLCi2 had the lowest value of maximum dose (Dmax) and V60Gy (Table 3). There were no significant differences obtained for the rectum, penile bulb, and both femoral heads with different types of MLCs. The bowel bag showed a significant difference with MLCi2 compared to Agility (FF or FFF).

Plan efficiency was evaluated with each type of MLCs via parameters such as MUs, time delivery (seconds), and NDC (Table 4). The present study found that Agility FFF required more MUs than the Agility FF and MLCi2 plans. Regarding the time of dose delivery, Agility FF and FFF plans significantly improved the time delivery compared to MLCi2 plans. Specifically, the actual time delivery was decreased with Agility MLC in both modes of energy by about 30% when compared to MLCi2. The quality of SIB-plan was assessed by measuring NDC for each type of MLC. For the NDC values close to 1 were obtained for all VMAT plans. However, Agility FF had a better plan quality in comparison to Agility FFF and MLCi2.

**Table 4:** Plan parameters of VMAT: number of MUs and delivery time for each type of multileaf collimator (MLC)

Parameters	VMAT technique on different types of MLC		
	Agility FF	Agility FFF	MLCi2
Monitor units (MUs)	1528.6 ± 267.2 (A)	1782.3 ± 349.5 (B)	1398 ± 230.3 (A)
Delivery time (Sec)	318.5 ± 21.1 (A)	332.8 ± 31.3 (A)	461.1 ± 90.65 (B)

For each parameter, there are statistically significant differences between different modes of energy (Agility FF, Agility FFF, and MLCi2) when indicated with different letters (A, B)

*P* ≤ 0.05 was defined as statistically significant

### IMRT dosimetric parameters of PTVs and OARs

Tables 5, 6 summarize the results of the second set off PTVs and OAR dosimetric parameters for the IMRT technique. For PTV60, the results revealed that there were no significant differences in D2% of Agility FF, FFF, and MLCi2, but that the values of D98%, D95%, D50%, and Dmean showed a significant difference in Agility FF and FFF compared to MLCi2. The PTV60 values of CI and HI for Agility FF and FFF were better than those of MLCi2 obtained with IMRT plans, but no significant differences were found between Agility FF and FFF.

**Table 5:** Dosimetric parameters of PTVs for IMRT plans (Mean ± SD, *n* = 10)

	IMRT technique on different types of MLC		
	Agility FF	Agility FFF	MLCi2
Parameters (PTV60)			
D2% (Gy)	63.2 ± 0.5)A)	63.5 ± 1.3 (A)	62.9 ± 0.7 (A)
D98% (Gy)	58.5 ± 1.34 (A)	58.7 ± 1.4 (A)	57.1 ± 1.1 (B)
D95% (Gy)	59.5 ± 0.8 (A)	59.6 ± 0.9 (A)	58.4 ± 0.7 (B)
D50% (Gy)	61.4 ± 0.4 (A)	61.8 ± 0.5 (A)	60.9 ± 0.5 (B)
D MAX (Gy)	65.4 ± 0.81 (A)	66.1 ± 1.5 (A)	65.1 ± 0.8 (A)
D min (Gy)	51.6 ± 4.63 (A)	50.9 ± 4.9 (A)	46.7 ± 6.6 (A)
D mean (Gy)	61.3 ± 0.4 (A)	61.7 ± 0.6 (A)	60.8 ± 0.5 (B)
Homogeneity (HI)	0.083 ± 0.02 (A)	0.08 ± 0.02 (A)	0.093 ± 0.01 (B)
Conformity (CI)	0.91 ± 0.1 (A)	0.93 ± 0.04 (A)	0.8 ± 0.08 (B)
Parameters (PTV44)			
D2% (Gy)	56.3 ± 1.03 (A)	56.6 ± 1.4 (A)	55.6 ± 0.89 (B)
D98% (Gy)	43.4 ± 0.8 (A)	43.6 ± 0.4 (A)	42.7 ± 0.69 (B)
D95% (Gy)	44.1 ± 0.5 (A)	44.2 ± 0.3 (A)	43.6 ± 0.5 (B)
D50% (Gy)	46.2 ± 0.2 (A)	46.6 ± 1.2 (A)	46 ± 0.2 (B)
D MAX (Gy)	63 ± 0.8 (A)	63.5 ± 1 (A)	63 ± 1.8 (A)
D min (Gy)	38 ± 2.4 (A)	38.5 ± 1.7 (A)	36 ± 2.8 (B)
D mean (Gy)	46.7 ± 0.2 (A)	46.7 ± 0.24 (A)	46.4 ± 0.2 (B)
Homogeneity (HI)	0.29 ± 0.03 (A)	0.29 ± 0.032 (A)	0.29 ± 0.03 (A)
Conformity (CI)	0.96 ± 0.03 (A)	0.96 ± 0.018 (A)	0.93 ± 0.03 (B)
NDC	0.97 ± 0.01 (A)	0.97 ± 0.01 (A)	0.96 ± 0.01 (B)

For each parameter (D2%, D98%, D95%, there are statistically significant differences between different modes of energy (Agility FF, Agility FFF, and MLCi2) when indicated with different letters (A, B)

*D2%* dose received by 2% of the PTV, *D98%* dose received by 98% of the PTV, *D95%* dose received by 95% of the PTV, *D50%* dose received by 50% of the PTV, *Dmax* maximum dose of PTV, *Dmin* minimum dose of PTV, *Dmean* average dose of PTV

*P* ≤ 0.05 was defined as statistically significant

**Table 6:** IMRT plans for organs at risk (OAR_S_) with dosimetric parameters

Parameters of OARs	IMRT technique on different types of MLC		
	Agility FF	Agility FFF	MLCi2
Bladder			
V60Gy < 25%	9.4 ± 2.4 (A)	9.7 ± 2 (A)	8 ± 2.9 (B)
V56Gy < 35%	16.1 ± 1.1 (A)	16.3 ± 1.7 (A)	15.2 ± 1.4 (B)
V52Gy < 50%	20.9 ± 12 (A)	20.8 ± 12.1 (A)	20.6 ± 12.7 (A)
Mean dose	36 ± 1.45 (A)	36 ± 1.4 (A)	37 ± 0.8 (B)
Maximum dose	64 ± 0.6 (A)	64.5 ± 1 (A)	64.3 ± 1.2 (A)
Rectum			
V60Gy < 15%	3.4 ± 3.1 (A)	4.5 ± 3.9 (B)	3.1 ± 3.7 (A)
V56Gy < 25%	8.4 ± 3.9 (A)	9.3 ± 5.3 (B)	8.4 ± 5.2 (A)
V52Gy < 35%	12.7 ± 4.3 (A)	13.8 ± 5.9 (A)	13.04 ± 5.73 (A)
Mean dose	34.2 ± 1.4 (A)	35.5 ± 1.5 (B)	36.1 ± 1.6 (B)
Maximum dose	63 ± 1.2 (A)	63.5 ± 1.8 (A)	62.9 ± 1.5 (A)
Femoral (MAX dose) < 45 Gy			
Right	39 ± 1.8 (A)	40.4 ± 2.3 (B)	40.2 ± 3.3 (B)
Left	38.8 ± 1.96 (A)	39 ± 2.67 (A)	38.5 ± 2.75 (A)
Bowel bag			
V45gy < 200 ml	71.3 ± 70.5 (A)	64.8 ± 66.1 (B)	61.7 ± 63.5 (B)
D(5 ml) < 60 Gy	47.4 ± 1.8 (A)	47 ± 1.7 (B)	47 ± 2.7 (B)
Penile bulb			
D (mean) < 45 Gy	22.5 ± 8 (A)	22.4 ± 8.3 (A)	22.95 ± 7.1 (A)

For each parameter, there are statistically significant differences between different modes of energy (Agility FF, Agility FFF, and MLCi2) when indicated with different letters (A, B)

OARs parameters: *VX Gy < X%* percent volume of OARs receiving a dose of x Gy in less than x% of the volume; *Dmax* maximum dose, *Dmean* average dose, *VX Gy < Xml* volume of OARs in milli litter (ml) receiving a dose of x Gy is less than x ml of the volume, *DX ml < X Gy* no more than X Gy received by X ml of the volume

*P* ≤ 0.05 was defined as statistically significant

For PTV44, D2% had the lowest value for MLCi2 as compared to Agility FF and FFF. However, Agility MLC (FF and FFF) demonstrated a statistically significant improvement for other dosmetric parameters (D98%, D95%, D50%, Dmin, Dmean, and CI) as compared to MLCi2.

The OARs (Table 6) met the criteria that had been set in the planning system with all plans using the IMRT technique with different types of MLCs and different modes of energy. V60 Gy and V56 Gy for bladder dose were improved with MLCi2 than Agility MLC, but the mean dose using FF and FFF was significantly lower than that using MLCi2. In addition, the rectum dose showed better values at V60 Gy and V56 Gy with Agility FF and MLCi2 than with Agility FFF, while the mean dose was significantly lower with Agility FF than with FFF and MLCi2. MLCi2 and Agility FFF had the lowest values for V45 and D (5 ml) for bowel bags compared to Agility FF. Delivery efficiency and SIB-plan quality were compared for all IMRT plans at different types of MLCs, and the results are presented in Table 7. Agility FFF and FF plans required more MUs in the IMRT technique than in the MLCi2 technique, but the delivery time was shorter for Agility FF and FFF compared to MLCi2. The actual delivery time was more than 11% lower for MLC Agility in IMRT plans than for MLCi2. SIB-plan quality was significantly improved for Agility FFF and FF as compared to MLCi2.

**Table 7:** Plan parameters (IMRT): number of MUs and delivery time with each type of MLCs

Parameters	IMRT technique on different types of MLC		
	Agility FF	Agility FFF	MLCi2
Monitor units (MUs)	1550.8 ± 257 (A)	1755.2 ± 266 (A)	1268.9 ± 130.2 (B)
Delivery time (sec)	398.7 ± 38.2 (A)	411.6 ± 45.4 (A)	447.1 ± 27.1 (B)

For each parameter, there are statistically significant differences between different modes of energy (Agility FF, Agility FFF, and MLCi2) when indicated with different letters (A, B)

*P* ≤ 0.05 was defined as statistically significant

## Discussion

Recently, the use of MLCs has become one of the most important innovations in radiation therapy, because it offers the required level of treatment while preserving normal tissues (Hong et al. 2014). Depending on leaf width, MLCs enable the planning system to produce high-quality plans with fewer segments, less monitoring units, and shorter time (Kantz et al. 2015). The results of this study show that certain MLC design parameters, such as leaf width and traveling speed, had an effect on several parameters such as required MUs, segments, and treatment time, allowing adjustment of each MLC design to achieve the best plan possible. Various favorable MLC types and energy modalities were found among agility MLCs as a result of this study.

Based on the technique used, the results of this study were divided into two groups, VMAT and IMRT procedures with Agility FF and FFF, which produced significantly better dosimetric results for PTV60 than for MLCi2. The CI and HI values for Agility in both modes of energy (FF and FFF) were enhanced. This is consistent with prior research that investigated the effect of MLC widths on tumour dose distribution using a variety of radiation modalities. For example, Chae et al. (2014) compared the target coverage and gradient index of two MLC widths (2.5 mm and 5 mm) for VMAT and IMRT procedures in the treatment of spinal lesions. They found that the lower leaf width (2.5 mm) enhanced the target coverage and gradient index. (Blümer et al. 2014) used VMAT to compare two types of MLCs (5 mm and 10 mm). They found that better HI and CI in the plan for 5 mm than for 10 mm MLCs. With the exception of the femoral head in prostate and anal cancer patients and the spinal cord in head and neck cancer (HNC) patients, the DVH results for OARs in all three cancers with different sites in VMAT plans using 10 mm MLCs were identical. Using the VMAT approach, (Lafond et al. 2013) evaluated the effect of leaf width between 10 and 4 mm for HNC patients. They also demonstrated that for 4 mm MLC, the CI and HI for PTV were increased by 4.7% and 7.9%, respectively. Also, the target coverage was enhanced with 4 mm MLC rather than with 10 mm in nasopharyngeal IMRT, but there was no benefit in terms of OAR avoidance (Wang et al. 2011). The CI was significantly improved using a small MLC width and the target volume coverage was higher as compared when using a larger MLC width (Jin et al. 2005); (Dvorak et al. 2005). Additionally, the results showed that both MLC agility techniques outperformed MLCi2 in terms of PTV coverage, whereas there was no significant difference between FF and FFF. These findings were consistent with (Sun et al. 2018) who observed no significant variations in target dose distributions for esophageal cancer between FF plans and FFF plans using the VMAT technique. Their results showed that smaller leaves (agility) can preserve OARs as well as large leaves, but PTV coverage increased with decreasing leaf width, which indicates that MLCi2 may achieve the limitations around OARs without decreasing PTV coverage.

Treatment delivery efficiency was greatly improved because the leaf speed was higher with agility MLC than with other modalities. These results were achieved employing agility VMAT and IMRT techniques, which were assisted by high-efficiency treatments that reduced treatment time even while delivering a high dose per fraction. It was reported that using modern radiotherapy technologies to reduce treatment duration resulted in greater compliance by patients and mild toxicity (Franco et al. 2021). Also, using Agility MLC during treatment may enhance patient comfort and reduce intra-fraction motion around organs.

The results of the present study demonstrate that, when compared to MLCi2, Agility reduced treatment time for VMAT and IMRT by 30% and 11%, respectively. When compared with the prescribed dose, Agility MLC delivered more MUs than MLCi2. To achieve a uniform dose distribution for SIB plans, the MUs in Agility were increased, which required an increase in segment numbers. Agility also involved a higher dose rate than MLCi2, therefore it required less time. However, it has been also shown that a number of parameters, including MUs, dose rate, and MLC movement speed, influence the delivery time as well. SIB-plan quality (as measured by the NDC factor) exhibited significant differences for Agility FF and FFF when compared with MLCi2 for both IMRT and VMAT plans. FFF required more MUs than FF to fulfill homogeneity and dose uniformity in PTVs. FFF has also been demonstrated to deliver a high dose rate, which helps in reducing treatment time by providing the highest dose per fraction for stereotactic body radiation (SBRT) (Sun et al. 2018).

## Conclusion

The width of MLCs plays a pivotal role in SIB-plan quality. MLC design can improve plan dosimetric parameters and overcome challenges to creating optimal treatment plans for prostate cancer with advanced radiotherapy modalities. For both radiotherapy techniques (IMRT and VMAT), the smaller width of MLCs (5 mm) always produced better PTV coverage, improved the dosimetric parameters for PTV, and reduced the dose delivery time for prostate cancer patients; due to better target coverage and better protection of OARs. Consequently, it is recommended to use 5 mm MLCs for prostate hypofractionated treatment. For IMRT, although OAR met the requirements, there were significant differences between FFF and FF in certain organs.

## Data Availability

All data generated or analyzed during this study are included in this published article (the raw data will be available in case required them from the authors).
